# Can we prevent ondansetron induced fatal ventricular tachycardia?

**DOI:** 10.4103/0253-7613.51352

**Published:** 2009-04

**Authors:** Onkar C. Swami

**Affiliations:** Manager - Medical Services, Emcure Pharmaceuticals Ltd, Rajiv Gandhi IT Park Phase I, MIDC, Hinjwadi Pune - 411 057, India. E-mail: Onkar.Swami@emcure.co.in

Sir,

We have read with interest the article ‘Ondansetron induced fatal ventricular tachycardia’ by Chandrakala *et al.,* published in the Indian Journal of Pharmacology, August 2008. We would like you to give us the opportunity to discuss some of our observations on the same.

Ondansetron is a racemic compound having one chiral centre. Racemic Ondansetron is a 50:50 mixture of two enantiomers, R-Ondansetron and S-Ondansetron. In one animal study (in dogs), effects of R-Ondansetron, S-Ondansetron and racemic Ondansetron on cardiac arrhythmias (cardiotoxicity) were evaluated. QTc interval was most prolonged among animals receiving S-Ondansetron and racemic Ondansetron and was shortest among animals receiving R-Ondansetron. Based on these results it was reported that R-Ondansetron has less cardiotoxicity than S-Ondansetron or racemic Ondansetron.[1]

Bodhankar *et al.* in 2006 studied the effect of racemic Ondansetron, R-Ondansetron and S-Ondansetron on QTc interval in electrocardiograms of rats. The Ondansetron and its enantiomers were administered IV in a dose of 3 mg/Kg and changes in QT and RR interval and heart rate were calculated. The results of this study showed that racemic Ondansetron and S-Ondansetron significantly prolonged QTc interval, while R-Ondansetron did not prolong QTc interval as compared to the vehicle treated group. They concluded that R-Ondansetron is safer on the heart. Authors reported that S-Ondansetron might have higher inhibitory effects on Bezold-Jarisch reflex and this might be the reason for QTc prolongation with S-Ondansetron and racemic Ondansetron.[2]

A multicentric, randomized, double-blind, parallel group, comparative study on efficacy and safety of R-Ondansetron 4 mg versus racemic Ondansetron 8 mg in 240 Indian patients with nausea and vomiting concluded that R-Ondansetron 4 mg BID was equally effective compared to racemic Ondansetron 8 mg BID with a slightly better safety profile compared to racemic Ondansetron.[3] This suggests that the S-Ondansetron component can be omitted without loss of efficacy. Efficacy and safety of R-Ondansetron solution was also studied in 410 pediatric patients confirming R-Ondansetron solution is safe and effective in pediatric patients with nausea and vomiting.[4]

From the above discussion it is clear that R-Ondansetron 4 mg alone is sufficient for its anti-emetic potential and is preferable without its counterpart – the S-isomer which can prolong the QTc. So switching from racemic Ondansetron to R-Ondansetron is a rational therapeutic approach. R-Ondansetron has been approved by the DCGI on 15^th^ April 2005 and is available as tablets (2 mg/4 mg), solution (1 mg/5 ml) and injection (1 mg/ml).
